# Maternal transmission of a mild Coffin–Siris syndrome phenotype caused by a *SOX11* missense variant

**DOI:** 10.1038/s41431-021-00865-2

**Published:** 2021-03-31

**Authors:** Britta Hanker, Gabriele Gillessen-Kaesbach, Irina Hüning, Hermann-Josef Lüdecke, Dagmar Wieczorek

**Affiliations:** 1 Ambulanzzentrum UKSH, Institut für Humangenetik, Universitätsklinikum Schleswig-Holstein, Lübeck, Germany; 2 Institut für Humangenetik, Universitätsklinikum Schleswig-Holstein, Lübeck, Germany; 3 Präsidium der Universität zu Lübeck, Lübeck, Germany; 4 Institut für Humangenetik, Universitätsklinikum Düsseldorf, Heinrich-Heine-Universität Düsseldorf, Düsseldorf, Germany

**Keywords:** ADHD, Autism spectrum disorders

## Abstract

Here we report for the first time on the maternal transmission of mild Coffin–Siris syndrome (CSS) caused by a *SOX11* missense variant. We present two sisters with intellectual disability and muscular hypotonia born to non-consanguineous parents. Cogan ocular motor apraxia was present in both sisters. Body measurements were in a normal range. The mother and both daughters showed hypoplastic nails of the fifth toes. A missense variant in *SOX11* [c.139 G > A; p.(Gly47Ser)] in both sisters and their mother was identified. Since 2014, variants in *SOX11* are known to cause mild CSS. Most described patients showed intellectual disability, especially concerning acquired language. All of them had hypoplastic nails of the fifth toes. It is of note, that some of these patients show Cogan ocular motor apraxia. The facial dysmorphic features seem not to be specific. We suggest that the combination of Cogan ocular motor apraxia, hypoplastic nails of fifth toes, and developmental delay give the important diagnostic clue for a variant in the *SOX11* gene (OMIM 615866, MR 27).

## Introduction

Coffin–Siris syndrome (CSS) was first described in 1970 by the pediatrician Grange S. Coffin and the radiologist Evelyn Siris [1]. CSS is characterized by aplasia or hypoplasia of the distal phalanx or nail of the fifth and additional digits and/or toes, intellectual disability, characteristic facial features, growth deficiency, microcephaly, hypertrichosis, and sparse scalp hair. The inheritance seems to be autosomal dominant. Variants in several genes encoding components of the BRG1- and BRM-associated factor (BAF) complex were identified. The BAF complex, one of the ATP-dependent chromatin remodeling complexes, mediates the opening and closing of chromatin [2]. Variants in multiple components of the BAF complex (*ARID1A*, *ARID1B*, *DPF2, SMARCA4*, *SMARCB1*, and *SMARCE1)* and PBAF complex (*ARID2*) have been implicated as causes of CSS [3–10].

The *SOX11* gene (OMIM 600898, SRY-BOX11) is located on chromosome 2p25.2. *SOX11* is part of the SRY box-related (SOX) sequences. They contain only one DNA-binding domain, and they bind to DNA in a sequence-specific manner. They act as potential transcription factors implicated in the developmental control of gene expression [11].

Here we describe a novel heterozygous missense *SOX11* variant in two sisters and her mother presenting with ocular motor apraxia and hypoplastic nails of fifth toes.

## Patients and methods

### Daughter 1

**Fig. 1:**
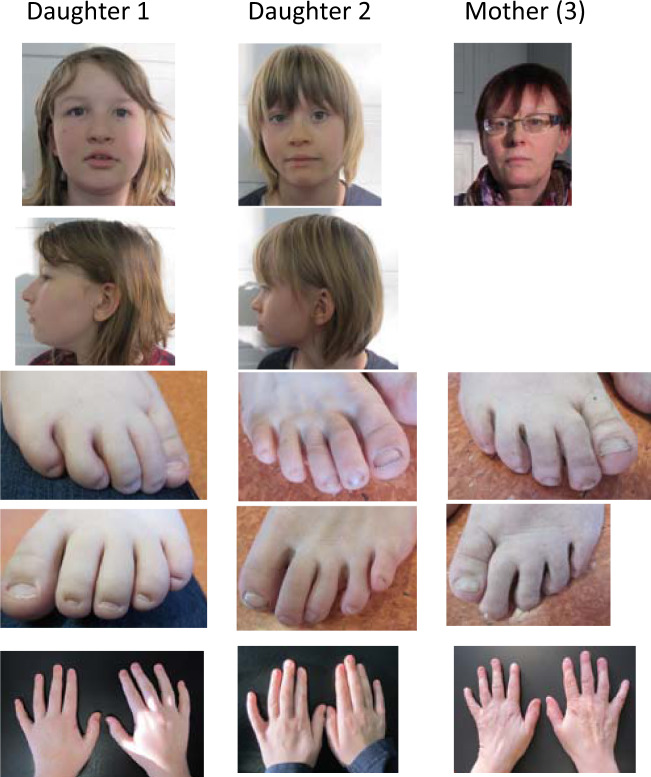
Clinical photographs of the family members. Daughter 1 and 2, mother (3). Facial features include well-defined eyebrows, short philtrum, and full lips (daughter 1). Hypoplastic fifth toenails were present in all patients. Permission has been obtained from the patient’s parents and mother (3) herself for presentation.

The first of two daughters was born of non-consanguineous parents after 40 weeks of gestation following an uncomplicated pregnancy and delivery. Birth weight (−1.4 SD), birth length (+0.6 SD), and head circumference (+0.1 SD) were in a normal range. Muscular hypotonia was noted after birth. The girl was able to walk alone at the age of 15–18 months. First words were spoken at the age of 12 months. During childhood, moderate cognitive impairment was apparent, she visited a special class for children with learning difficulties. Dysmorphic features included short philtrum, thick lips, sunken eyes, and strabism (Fig. 1). Low-set ears were present. She had no hirsutism but sparse scalp hair. In addition, Cogan ocular motor apraxia was present. Brain MRI was normal. Karyotyping and array-CGH gave normal results. Fragile-X-syndrome was negative by CGG-repeat expansion. Reexamination at age of 12 ^7^/_12_ years showed normal body measurements (weight (0 SD), height (−0.8 SD), and head circumference (+1.2 SD)). Hypoplastic nails of the fifth toes were present suggesting the tentative diagnosis of a phenotype consistent with CSS. Sanger sequencing of *ARID1B* gave normal results.

### Daughter 2

The second child was born 4 years later after 30^+2^ weeks of gestation. Delivery was preterm due to intrauterine growth restriction. Due to advanced maternal age, an amniotic karyotyping was performed showing a normal female karyotype. Birth weight (−1.2 SD), birth length (−0.9 SD), and head circumference (−0.8 SD) were in a normal range. She was able to walk at the age of 18 months. First words were spoken at the age of 14 months. She had a duplicated kidney on the left side. The ophthalmological assessment confirmed Cogan’s ocular motor apraxia and strabism.

At reexamination, at age of 8 ^3^/_12_ years, hypoplastic nails of the fifth toes were noticed. Facial dysmorphic features consisted of a depressed nasal bridge and full lips (Fig. 1). Her body measurements were still in the normal range (weight (−0.5 SD), height (+0.6 SD), and head circumference (−0.8 SD)). Overall, she was more severely affected. She had more severe speech impairment and difficulties in speech comprehension than her sister.

### Mother (3)

The mother, who had a history of learning difficulties in childhood and also went to a special class for learning difficulties, finally made graduation from modern secondary school. In childhood, she had a history of nephritis and meningitis. She shows an iris coloboma on the right side and was surgically treated because of a bilateral cataract. She also showed hypoplastic nails of the fifth toes (Fig. 1). Bilateral syndactyly of toes II-IV was also noticed. Her head circumference was (−1.9 SD) and her height (−0.8 SD). Her bodyweight was in a normal range.

The phenotypes of the family members are summarized in Table 1.

**Table 1:** Clinical overlap of our family and the six previously reported patients with *SOX11* variants.

	1	2	3	4	5	6	7	8	9	Total
	Tsurusaki		Hempel			Okamoto	our family			
Origin	Japanese	Indian	nk	nk	nk	Japanese	German	German	German	
Gender	Female	Female	Female	Male	Male	Male	Female	Female	female	
Patient number	1	2	8	9	10	1	Daughter 1	Daughter 2	Mother (3)	
*Mutation*										
c.	c.347 A > G	c.178 T > C	c.359 C > A	c.150 G > C	c.87 C > A	c.305 C > T	c.139 G > A	c.139 G > A	c.139 G > A	
p	p.(Tyr116Cys)	p.(Ser60Pro)	p.(Pro120His)	p.(Lys50Asn)	p.(Cys29*)	p.(Ala102Val)	p.(Gly47Ser)	p.(Gly47Ser)	p.(Gly47Ser)	
*Growth and feeding*										
Gestation	38	40	41	40		41	40	31	nk	
Prenatal growth deficiency	✚	✚					⎯	✚		
Birth weight (g)	2340 (−1.9 SD)	1750 (−4 SD)	3118	3500	4080	2960	2875 (−1.4 SD)	1055 (−1.2 SD)	nk	
Birth length (cm)	45 (−2.2 SD)						53 (+0.6 SD)	37 (−0.9 SD)	nk	
Birth OFC (cm)	30.5 (−1.8 SD)						35 (+0.1 SD)	27 (−0.8 D)	nk	
Postnatal growth										
*Growth deficiency*	**✚**	**✚**	**✚**	**✚**		**✚**	**⎯**	**⎯**		**5 (9)**
Age	10 year	17 year	12 year, 6 months	11 years	6 year, 10 months	5 year	12 year, 7 months	8 year, 3 months	51 year	
Weight (kg)	20.1 (−1.8 SD)	31.3 (−3 SD)	12.15	18.45	29	14.5 (−1.7 SD)	48.5 (0 SD)	25 (−0.5 SD)	nr	
Height (cm)	119 (−2.8 SD)	141 (−5 SD)	89.4	109.4	124.5	97 (−2.9 SD)	153 (−0.8 SD)	134 (+0.6 SD)	163	
OFC (cm)	47.3 (−3.3 SD)	50.5 (−4.5 SD)	46.5	48.2	54	48.5 (−1.5 SD)	55.5 (+1.2 SD)	51 (−0.8 SD)	53 (−1.9 SD)	
Sucking/feeding difficulty	✚	⎯	✚	✚		✚	⎯	⎯	nk	4 (9)
*Craniofacial features*										
*Microcephaly*	**✚**	**✚**	**✚**	**✚**		**⎯**	**⎯**	**⎯**	**⎯**	**4 (9)**
Midface hypoplasia	✚	⎯				✚	⎯	⎯	⎯	
Short palpebral fissure	✚						⎯	⎯	⎯	
Hypertelorism	✚						⎯	⎯	⎯	
Flat nasal bridge	✚	⎯	✚				⎯	✚	⎯	
Shortened nose	✚					✚	⎯	⎯	⎯	
Upturned nostrils	✚						⎯	⎯	⎯	
Longnose		✚					⎯	⎯	⎯	
Hypoplastic alae nasi		✚					⎯	⎯	⎯	
Prominent columella		✚					⎯	⎯	⎯	
Short philtrum	✚	⎯		✚		✚	✚	⎯	⎯	4 (9)
Open mouth	✚						⎯	⎯	⎯	
*Full lips*	**✚**	**⎯**	**✚**		**✚**	**✚**	**✚**	**✚**	**⎯**	**6 (9)**
Sparse scalp hair	⎯	✚(mild)				⎯	✚	⎯	⎯	
Abundant scalp hair	✚						⎯	⎯	⎯	
Hypertrichosis	✚	✚				✚	⎯	⎯	⎯	
Thick eyebrow	⎯	⎯					⎯	⎯	⎯	
Long eyelashes	✚	⎯				⎯	⎯	⎯	⎯	
Sunken eyes			✚				✚	⎯	✚	
Arched eyebrows	✚	✚			✚	✚	⎯	⎯	⎯	4 (9)
Ptosis		⎯					⎯	⎯	⎯	
Low set ears	✚	✚					✚	⎯	⎯	
Thin upper vermilion							⎯	⎯	⎯	
Everted lower lip	✚	✚					⎯	⎯	⎯	
Wide mouth	⎯	⎯	✚		✚		⎯	⎯	⎯	
Micrognathia		✚					⎯	⎯	⎯	
Cleft palate	⎯	⎯					⎯	⎯	⎯	
*Skeletal-limb features*										
Hypoplastic fifth fingernails	✚	✚				✚	⎯	⎯	⎯	
Hypoplastic distal phalanges	✚	✚					⎯	⎯	⎯	
*Hypoplastic fifth toenails*	**✚**	**✚**	**✚**	**✚**	**✚**	**✚**	**✚**	**✚**	**✚**	**9 (9)**
2–3 syndactyly fingers				✚	✚		⎯	⎯	⎯	
Syndactyly toes							✚	✚	✚	
Clinodactyly		✚	✚	✚	✚	✚	✚	✚	⎯	7 (9)
Hearing and vision										
Hearing impairment	⎯	⎯		✚			⎯	⎯	⎯	
Visual impairment	✚	⎯		Hypermetropia			Strabism	Strabism	✚	
*Cogan ocular motor apraxia*			**✚**		**✚**		**✚**	**✚**	**nk**	**4 (9)**
*Neurology*										
Hypotonia	✚	⎯					✚	✚	⎯	
Autism			✚				⎯	⎯	⎯	
Seizures	⎯	⎯	Absence s.				⎯	⎯	⎯	
Structural CNS abnormalities			⎯		Hypoplasia cerebellar vermis	⎯	⎯	nk	Coloboma iris	
*Development and Intelligence*										
*Developmental delay*	**✚**	**✚(mild)**	**✚**	**✚**	**✚**	**✚**	**✚**	**✚**	**✚**	**9 (9)**
Severe			✚							
Moderate						✚				
Mild	✚	✚					✚	✚		
Sit (month)	11	Normal range							nk	
Walk (month)	23	Normal range	30	24	16	27	17	18	nk	
*Speech impairment*		**✚**	**✚**	**✚**	**✚**	**✚**	**✚**	**✚**		**7 (9)**
No words		✚	✚							
First words (month)	19			36			12	14		
*Other*										
Renal abnormalities	Left small kidney	Bilateral malrotated kidneys					⎯	Duplicated kidney left		
Cardiac anomaly						Coartation VSD				

*y* years, *m* months, *nr* normal range, *nk* not known, *OFC* occipito-frontal circumference, + feature present, ⎯ feature absent.

(1) 1 + 2 Adapted from [5, 12], reference sequence NM_003108.3.

(2) 3–5 Adapted from [13], reference sequence NM_003108.3.

(3) 6 Adapted from [14], reference sequence NM_003108.

Written informed consent was obtained from the parents for participation in this study. The study was performed according to the Declaration of Helsinki protocols. DNA from peripheral blood lymphocytes was obtained and extracted by standard extraction procedures.

NGS panel-analysis of the genes *SOX11*, *ARID1A*, *ARID1B*, *ARID2*, *SMARCA4*, *SMARCB1*, and *SMARCE1* and *PHF6* was performed in the two sisters. The mean coverage overall targets of the above genes were 297x (min: 35x, max 606x) for daughter 1 and 336x (min: 36, max 719x) for daughter 2. The presence of the variant was confirmed in daughter 1, 2, and the mother (3) by Sanger sequencing. The variant was absent in the DNA of the father of the girls. The variant was submitted to the database of LOVD (URL: http://www.LOVD.nl/SOX11, DB-ID SOX11_000021, Individual ID 00306973).

## Results

In this study, we identified a *SOX11* missense variant ([GRCh38/hg38] chr2:g.5,692,860 G > A; NM_003108.3: c.139 G > A; NP_003099.1: p.(Gly47Ser)) in the mother and her two daughters. The variant has not been described before in HGMD *prof*., LOVD and ClinVar. Four in silico-Tools (PROVEAN, score −5.464; SIFT, score 0.01; Polyphen-2, scores HumDiv 1.000 and HumVar 0.999 and MutationTaster, “Disease Causing”) predicted the variant as probably pathogenic. No pathogenic variant was found in the other known CSS genes. The in silico CADD score was 31 (Request: Chromosome 2, position 5692860, CADD GRCh38-v1.6).

## Discussion

Here we identified for the first time a maternal transmission of a *SOX11* variant.

In 2014, Tsurusaki et al. reported for the first time on de novo variants in *SOX11* to cause CSS [12]. In 2016, Hempel et al. reported three patients with de novo *SOX11* variants and seven with a deletion in 2p25 including *SOX11* [13]. Okamoto et al. described one patient with a novel *SOX11* variant who showed coarctation of the aorta in addition to clinical features of CSS [14]. Six missense variants and one nonsense variant were identified in *SOX11* [12–14]. All missense variants are located in the HMG DNA binding domain. Our variant was located at the beginning of the HMG domain (SSF47095). Variants outside the HMG domain have been described which result in ocular malformations without developmental delay. In this work, we focused on the descriptions of mild CSS due to *SOX11* variants. A summary of the clinical signs is shown in Table 1.

The phenotype of the present cohort of patients with clinically diagnosed CSS variants in *SOX11* is to a larger extent homogeneous (Table 1). This can be partly explained by the choice of patients for molecular analysis. The two patients in the study of Tsurusaki et al. [12] had the suspicion of CSS. Two of the three patients with variants in *SOX11* presented by Hempel et al. [13] were part of the deciphering developmental disorders (DDD) study [15]. The third patient was identified by exome sequencing via the Genetics Of Structural Brain Abnormalities And Learning Disabilities Study (Wales Research Ethics Committee 12/WA/001 [16]). Hempel et al. [13]. reported that none of their cohorts had a clinical diagnosis of CSS before genetic analysis. But in retrospect, all individuals had clinical features compatible with CSS. The phenotype of all described patients with *SOX11* variants (in the HMG domain) shows that 100 % of the individuals have developmental delay and hypoplastic fifth toenails. There is a variation in the degree of developmental delay. Seven of them had delays in an acquired language.

The majority of patients present with growth deficiency. The facial characteristics can be quite different. There are no obvious distinctive facial features but short philtrum, full lips, and arched eyebrows are present in nearly half of the cases.

Ocular abnormalities were present in seven patients. One patient had hypermetropia another one had vision problems and three had a squint. Vision problems have not been specified (patient 1 from the study of Tsurusaki). The mother (3) of our family had iris coloboma and cataracts. Remarkably, four of the nine patients show Cogan ocular apraxia. Cogan oculomotor apraxia can occur isolated, in the combination with neurological findings, and as part of a syndrome (e.g., Joubert syndrome, Gaucher disease type 3, ataxia-telangiectasia). The number of undetected cases of Cogan ocular apraxia might be much higher because symptoms improve throughout childhood. It might be possible that the mother of our family had oculomotor apraxia during childhood. And in the study of Tsurusaki, the vision problems of patient 1 have not been specified. Possibly this patient had Cogan ocular apraxia, too.

Variants in *SOX11* seem to be a rare cause of mild CSS. We support the hypothesis that variants in BAF complex genes and *SOX11* result in a mild CSS phenotype, providing strong support for the BAF complex and SOX11 function in a common pathway, play an important role in human brain development. Cogan ocular apraxia in combination with developmental delay and hypoplastic nails of fifth toes might be the important diagnostic clue for recognizing patients with the variant in *SOX11*.

Regarding the in silico prediction for the missense variant and its Mendelian segregation pattern in the family in a dominant manner we suggest this variant to be causative for the clinical features.

## References

[1] Coffin GS, Siris E (1970). Mental retardation with absent fifth fingernail and terminal phalanx. Am J Dis Child.

[2] Hargreaves DC, Crabtree GR (2011). ATP‐dependent chromatin remodeling: genetics, genomics and mechanisms. Cell Res.

[3] Kosho T, Miyake N, Carey JC (2014). Coffin–Siris syndrome and related disorders involving components of the BAF (mSWI/SNF) complex: historical review and recent advances using next generation sequencing. Am J Med Genet C Semin Med Genet.

[4] Tsurusaki Y, Okamoto N, Ohashi H, Kosho T, Imai Y, Hibi‐Ko Y (2012). Mutations affecting components of the SWI/ SNF complex cause Coffin–Siris syndrome. Nat Genet.

[5] Tsurusaki Y, Okamoto N, Ohashi H, Mizuno S, Matsumoto N, Makita Y (2014). Coffin–Siris syndrome is a SWI/SNF complex disorder. Clin Genet.

[6] Santen GW, Aten E, Sun Y, Almomani R, Gilissen C, Nielsen M (2012). Mutations in SWI/SNF chromatin remodeling complex gene ARID1B cause Coffin–Siris syndrome. Nat Genet.

[7] Van Houdt JK, Nowakowska BA, Sousa SB, van Schaik BD, Seuntjens E, Avonce N (2012). Heterozygous missense mutations in SMARCA2 cause Nicolaides–Baraitser syndrome. Nat Genet.

[8] Wieczorek D, Bogershausen N, Beleggia F, Steiner‐Haldenstatt S, Pohl E, Li Y (2013). A comprehensive molecular study on Coffin–Siris and Nicolaides–Baraitser syndromes identifies a broad molecular and clinical spectrum converging on altered chromatin remodeling. Hum Mol Genet.

[9] Bramswig NC, Caluseriu O, Lüdecke HJ, Bolduc FV, Noel NC, Wieland T (2017). Heterozygosity for ARID2 loss-of-function mutations in individuals with a Coffin–Siris syndrome-like phenotype. Hum Genet.

[10] Vasileiou G, Vergarajauregui S, Endele S, Popp B, Büttner C, Ekici AB (2018). Mutations in the BAF-complex subunit DPF2 are associated with Coffin–Siris syndrome. Am J Hum Genet.

[11] Jay P, Goze C, Marsollier C, Taviaux S, Hardelin J-P, Koopman (1995). The human SOX11 gene: cloning, chromosomal assignment and tissue expression. Genomics.

[12] Tsurusaki Y, Koshimizu E, Ohashi H, Phadke S, Kou I, Shiina M (2014). De novo SOX11 mutations cause Coffin– Siris syndrome. Nat Commun.

[13] Hempel A, Pagnamenta AT, Blyth M, Mansour S, McConnell V, Kou I (2016). Deletions and de novo mutations of SOX11 are associated with a neurodevelopmental disorder with features of Coffin–Siris syndrome. J Med Genet.

[14] Okamoto N, Ehara E, Tsurusaki Y, Miyake N, Matsumoto N Coffin-Siris syndrome and cardiac anomal with a novel SOX11 mutation. Congenit Anom (Kyoto) 2017; Aug. 10.1111/cga.12242 28787104

[15] Wright CF, Fitzgerald TW, Jones WD, Clayton S, McRae JF, van Kogelenberg M (2015). Genetic diagnosis of developmental disorders in the DDD study: a scalable analysis of genome-wide research data. Lancet.

[16] Pagnamenta AT, Howard MF, Wisniewski E, Popitsch N, Knight SJ, Keays DA (2015). Germline recessive mutations in PI4KA are associated with perisylvian polymicrogyria, cerebellar hypoplasia and arthrogryposis. Hum Mol Genet.

